# A novel combination of corneal confocal microscopy, clinical features and artificial intelligence for evaluation of ocular surface pain

**DOI:** 10.1371/journal.pone.0277086

**Published:** 2022-11-01

**Authors:** Gairik Kundu, Rohit Shetty, Sharon D’Souza, Pooja Khamar, Rudy M. M. A. Nuijts, Swaminathan Sethu, Abhijit Sinha Roy

**Affiliations:** 1 Department of Cornea and Refractive surgery, Narayana Nethralaya, Bangalore, India; 2 Department of Cataract and Refractive surgery, Narayana Nethralaya, Bangalore, India; 3 Department of Ophthalmology, Maastricht University Medical Center, Maastricht, The Netherlands; 4 GROW Research Laboratory, Narayana Nethralaya Foundation, Bangalore, India; 5 Imaging, Biomechanics and Mathematical Modeling Solutions, Narayana Nethralaya Foundation, Bangalore, India; University of Pecs, HUNGARY

## Abstract

**Objectives:**

To analyse various corneal nerve parameters using confocal microscopy along with systemic and orthoptic parameters in patients presenting with ocular surface pain using a random forest artificial intelligence (AI) model.

**Design:**

Observational, cross-sectional.

**Methods:**

Two hundred forty eyes of 120 patients with primary symptom of ocular surface pain or discomfort and control group of 60 eyes of 31 patients with no symptoms of ocular pain were analysed. A detailed ocular examination included visual acuity, refraction, slit-lamp and fundus. All eyes underwent laser scanning confocal microscopy (Heidelberg Engineering, Germany) and their nerve parameters were evaluated. The presence or absence of orthoptic issues and connective tissue disorders were included in the AI. The eyes were grouped as those (Group 1) with symptom grade higher than signs, (Group 2) with similar grades of symptoms and signs, (Group3) without symptoms but with signs, (Group 4) without symptoms and signs. The area under curve (AUC), accuracy, recall, precision and F1-score were evaluated.

**Results:**

Over all, the AI achieved an AUC of 0.736, accuracy of 86%, F1-score of 85.9%, precision of 85.6% and recall of 86.3%. The accuracy was the highest for Group 2 and least for Group 3 eyes. The top 6 parameters used for classification by the AI were microneuromas, immature and mature dendritic cells, presence of orthoptic issues and nerve fractal dimension parameter.

**Conclusions:**

This study demonstrated that various corneal nerve parameters, presence or absence of systemic and orthoptic issues coupled with AI can be a useful technique to understand and correlate the various clinical and imaging parameters of ocular surface pain.

## Introduction

Ocular pain and chronic discomfort are generally reported by patients presenting to the ophthalmology outpatient clinic nowadays [1]. Successful pain management requires a comprehensive evaluation of various contributing factors. Hence, there is a need for improved understanding of ocular pain to manage these patients better. Symptoms could range from specific ones such as light sensitivity, burning, stinging, watery eyes, blurry vision to non-specific eye pain and inability to focus. Such issues are on the rise predominantly among younger subjects due to an increase in screen times and dependence on gadgets [2]. Since the beginning of the pandemic, home-based longer working hours on computers and increased use of face masks are contributing to a surge in patients presenting with non-specific ocular pain or discomfort [3].

Dry eye is one of the most common causes for ocular surface pain presenting to the clinic. However, there can be many other causes as well. These symptoms are described in TFOS DEWS II and can affect patients’ quality of life [4]. A significant problem facing clinicians is the management of patients whose symptoms are disproportionately greater than signs. Although typical features such as corneal staining, low tear break up time (TBUT), decreased Schirmer levels or meibomian gland dysfunction can help in classifying these patients, they may not be seen in all patients. Clinically unrecognisable inflammation in tears or corneal sub epithelial nerve plexus, neuropathic aetiologies or nutritional deficiencies (Vitamin D and B12) are possible contributors to dry eye associated ocular surface pain [5, 6]. Binocular vision issues related to convergence, fusion and accommodation are on rise among young people due to prolonged near work and screen times, and can lead to non-specific ocular surface pain and mimic dry eye symptoms [7].

The successful and comprehensive management of such pain and discomfort requires a thorough understanding of the role played by each of these contributing factors. Research on *in vivo* confocal microscopy (IVCM) has identified several changes in the sub-basal corneal nerves and linked it to changes in various corneal inflammatory mediators in patients with ocular surface pain [8]. While there are multiple parameters known to affect ocular surface pain, the role of these individual factors or as a combination is not well understood. As there are multiple corneal (sub-basal) nerve parameters involved, it is difficult to clearly identify the exact features which are significantly contributing to the pain. Interestingly there have been no studies linking artificial intelligence (AI) and these various changes at the sub-basal nerves using IVCM to ocular surface pain. Approaches based on machine learning have achieved excellent performance in analysis of medical images [9, 10]. Therefore, the objective of this study was to develop an AI model to link multiple corneal sub-basal nerve parameters using IVCM and demographic features with ocular surface pain to enable us to identify, understand and correlate these features more comprehensively.

## Methods

This was a retrospective analysis of patients presenting with ocular surface pain who visited the outpatient department of the Narayana Nethralaya eye hospital. The study was approved by the Narayana Nethralaya ethics committee (Reference number: C/2021/07/02). The study followed the tenets of the Declaration of Helsinki. A total of 300 eyes of 151 patients were evaluated. These included 240 eyes of 120 patients with the primary symptoms of ocular surface pain or discomfort and a control group of 60 eyes of 31 patients with no symptoms of ocular surface pain or discomfort and no clinically visible ocular signs on examination. Detailed clinical and systemic history were acquired. Ocular examination included visual acuity, refraction, slit-lamp and fundus imaging. In addition, non-strabismic binocular vision abnormalities and accommodation related problems which can lead to ocular surface pain or discomfort were also evaluated [7]. Patients using contact lenses, diagnosed with ocular infection in the last three months, uveitis and ocular trauma were excluded. Patients who had undergone any ocular surgery or were on any topical medication were also excluded. The presence of systemic/connective tissue disorders was an additional exclusion criteria for the control group.

### Assessment of ocular discomfort

The discomfort was assessed using the ocular surface disease index (OSDI) [11, 12]. An OSDI less than 12 was considered as normal, between 13 and 23 as mild, between 24 and 32 as moderate and greater than 32 as severe discomfort [11, 12]. Since dry eye disease (DED) is one the most common causes for ocular surface pain [13], a detailed dry eye evaluation including Schirmer’s test with and without topical anaesthetic, tear film breakup time (TBUT) and fluorescein staining (corneal and conjunctival) were done. TBUT and Schirmer test were graded as normal, mild, moderate and severe as described in Table 1 [4, 14].

**Table 1:** Classification and grading based on Tear film break up time (TBUT) and Schirmer’s test.

Grade	TBUT^a^ (seconds)	Schirmer’s test (mm/5min)
Normal	>10	>10
Mild	7–9	7–9
Moderate	5–7	5–7
Severe	<5	<5

^a^ Tear film break up time

### Orthoptic evaluation

A detailed orthoptic evaluation was done to assess abnormalities of the eye muscles that prevent normal binocular vision and may lead to ocular surface pain [15]. It included near point of convergence (NPC) and near point of accommodation (NPA). The normal cut-off for NPC was 8 cm for an accommodative target. The NPA changes with age and hence the cut-off value was considered as 18 –(0.3 × age) [15]. Other parameters measured were negative relative accommodation (NRA) which was the indirect assessment of positive fusional vergence and positive relative accommodation (PRA) for negative fusional vergence. The normal cut-off for NRA and PRA was +2.50D and -2.50D, respectively [15]. The negative fusional vergence (NFV) and positive fusional vergence (PFV) were also assessed as per standard protocol [15] and their measurements were recorded as break/recovery. The normal cut off for positive fusional vergence (base–out prism) in Dioptres for distance was 11/7 and for near was 19 /14. Similarly, negative fusional vergence (base–in prism) in Dioptres for distance was 7/4 and near was 13/10 [15]. These tests indicated possible accommodative and convergence related problems leading to ocular surface pain and were documented as present or absent for use in the AI model [7, 15].

### *In vivo* confocal microscopy

As corneal nerve related alterations are known to be associated with ocular surface pain, IVCM was done for all patients. They underwent laser scanning IVCM using the Rostock Corneal Module/Heidelberg Retina Tomograph II (Heidelberg Engineering, Germany). The section mode was used to scan the central cornea and two-dimensional digital images with an image size of 400×400 μm, a lateral digital resolution of 1 μm/pixel and a depth resolution of 2 μm/pixel were obtained. The various corneal nerve parameters studied using confocal microscopy were related to the sub-basal nerve plexus of the cornea and three high-quality sub-basal nerve plexus images were selected from each eye based on a previously defined standardised image selection protocol [16, 17]. The various corneal sub-basal nerve plexus parameters included were the nerve fibre length (CNFL), nerve fibre density (CNFD), nerve branch density (CNBD), nerve fibre total branch density (CTBD), nerve fibre area (CNFA), nerve fibre width (CNFW), nerve fractal dimension (CNFrD). In addition to these sub-basal nerve plexus parameters, features such as dendritic cell density, microneuromas and beading of nerves were also included in the AI analyses (Table 2) [18, 19]. The Fractal Dimension consisted of a nerve fibre detection step based on a machine learning method [20]. The nerve fibre fractal dimension measures the structure complexity as a ratio of the change in detail to the change in scale [21]. The automated corneal nerve fibre fractal dimension (CNFrD) measurement is now included in an automated nerve fibre quantification software, which is freely available from University of Manchester portal [22].

**Table 2:** Various parameters included in the artificial intelligence model.

Parameter group	Parameter Studied	Description
Confocal imaging parameters	CNFA	Corneal nerve fibre area per square millimetre
	CNFL	Corneal nerve fibre length in millimetres per square millimetre
	CNFD	Corneal nerve fibre density per square millimetre
	CNFW	Corneal nerve fibre width per square millimetre
	CNBD	The corneal nerve branch density per square millimetre
	CNFrD	The total branch density per square millimetre
	Total, Immature and Mature	Dendritic Cell Density-Quantification of hyper-reflective cells seen on confocal imaging
	Microneuroma	Specific feature seen in confocal microscopy-terminal enlargements of sub-basal corneal nerve
	Beading of nerves	Specific feature seen in confocal microscopy-foci of hyperreflective points and enlargements on corneal nerves
Orthoptics related issues	Presence or absence based on orthoptic evaluation.	
Systemic issues	Presence or absence based on blood investigations.	

**Fig 1:**
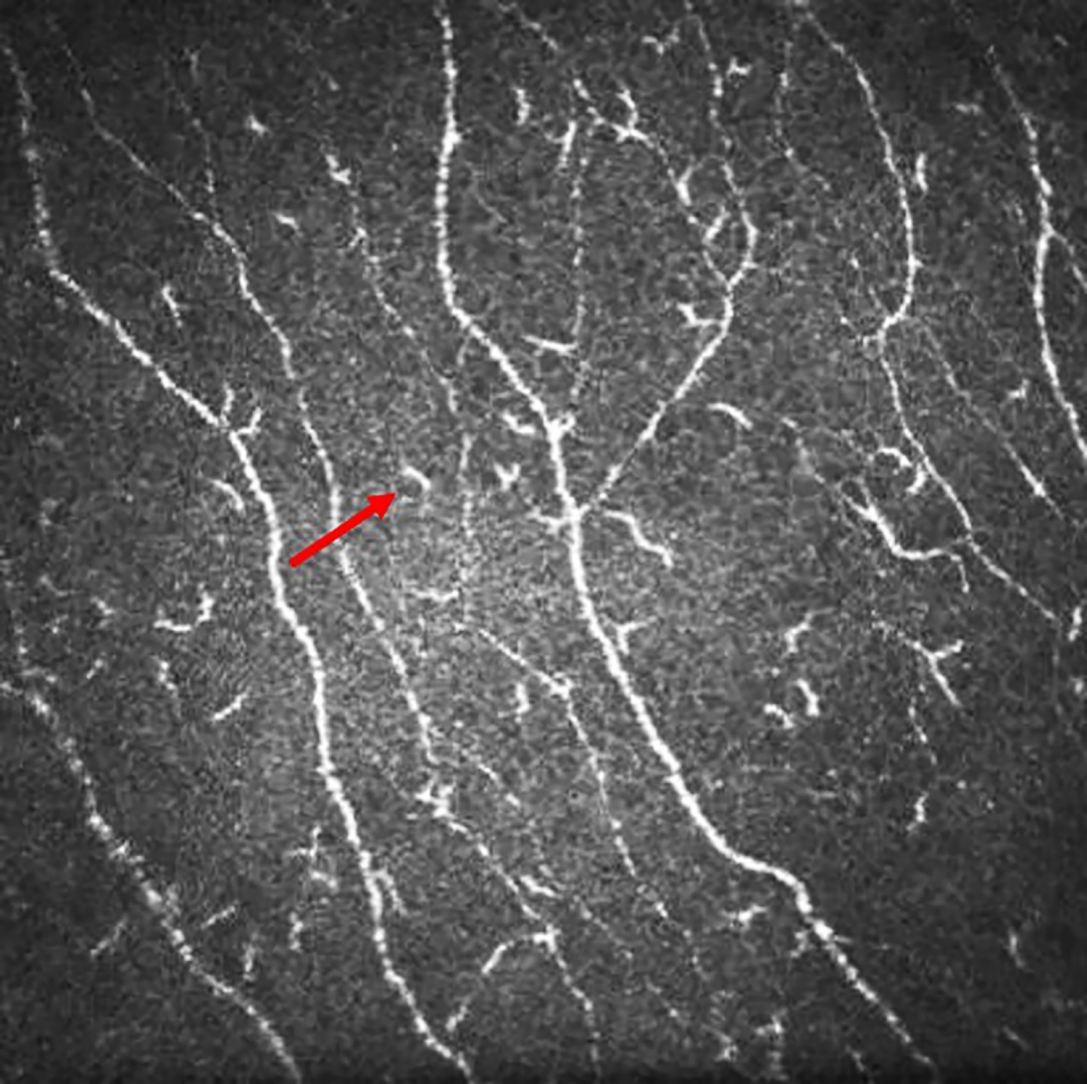
*In vivo* confocal image of the cornea showing sub-basal nerves with mature and immature dendritic cells. Red arrow indicates an immature dendritic cell.

**Fig 2:**
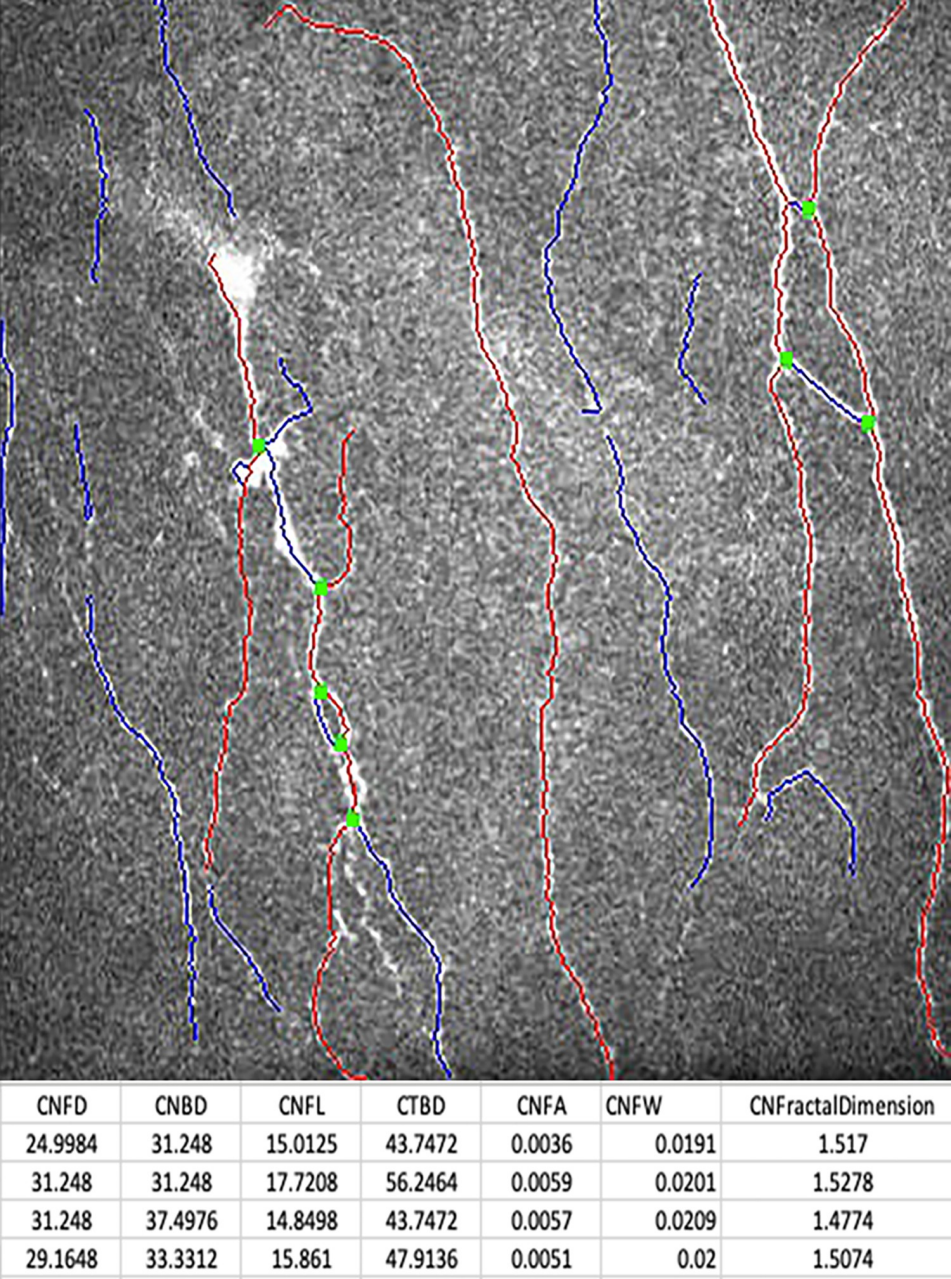
*In vivo* confocal microscopy image with superimposed software-aided nerve tracing and quantification of nerve length (CCMetrics).

**Fig 3:**
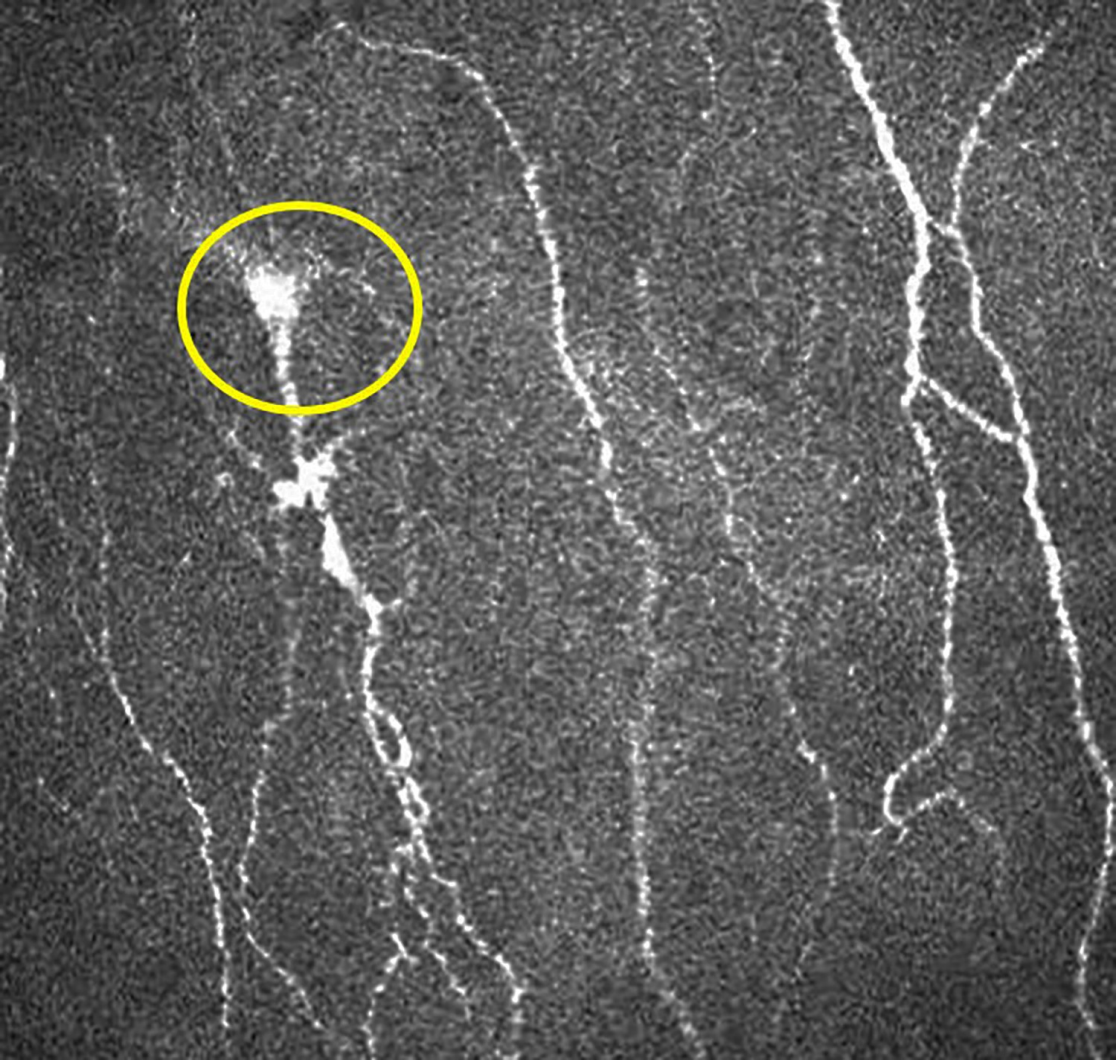
*In vivo* confocal microscopy of the cornea showing microneuroma indicated within the yellow circle.

The dendritic cell (DC) density was quantified from the same image frames used to quantify the sub-basal nerve plexus. The number of highly reflective cells with (mature) and without (immature) dendriform structures (Fig 1) were counted manually using the branch density quantification feature of the CCMetrics software (University of Manchester, Manchester, UK) (Fig 2). The density was derived as the number of cells in the area (mm^2^) of frame assessed [18, 19]. These cells were the Langerhan cells which were defined as bright dendritic structures in the images of sub-basal corneal nerves. Mature dendritic cells were antigen-presenting dendritic cells identified morphologically by a bright, reflective, slender cell body often having multiple long arm-like processes (dendrites) extending out from the main cell body, with an approximate total end-to-end length exceeding 25 to 45 μm or even longer. Immature dendritic cells were identified as small, reflective cell bodies without discernible dendrites or short dendrites with a total end-to-end length less than 25 μm [23, 24]. The microneuroma in the nerve plexus were identified (Fig 3) as described in recent studies [25, 26]. The beading of nerves on confocal imaging were the foci of hyper-reflective points, which were axon enlargements with a collection of mitochondria. They protruded slightly from the main truck and represented a natural response following nerve damage [27].

### Assessment of systemic status

As dry eye is one of the most common causes of ocular surface pain [14], systemic conditions, which can lead to dry eyes and ocular surface pain, were also investigated. Blood investigations to rule out connective tissue orders such as rheumatoid arthritis, systemic lupus erythematosus and Sjögren’s syndrome were done as per standard protocols and their presence or absence was documented for use in the AI [28].

All the eyes were classified into groups based on their ocular surface pain or discomfort symptoms (D) and signs (S). Group 1 (S^+/-^D^++^) included 76 eyes in whom the discomfort/symptom grade was higher than the grade/severity of signs. Group 2 (S^+^D^+^) included 131 eyes with similar grades of discomfort/symptoms and signs. Group 3 (S^+^D^-^) included 33 eyes without discomfort / symptoms (OSDI score: <12) but presented with signs (TBUT: <10 secs and/or Schirmer’s test 1: <10 mm / 5 mins). Group 4 (S^-^D^-^) included 60 eyes without discomfort / symptoms (OSDI score: <12) and no clinical signs (TBUT: ≥ 10 secs, Schirmer’s test 1: ≥ 10 mm / 5 mins). Hence, Group 4 subjects were presumed as normal or control eyes.

### AI model design

All the parameters of the four groups as mentioned above were evaluated using an AI model—the random forest (RF) classifier. RF classifier is fundamentally an ensemble of many decision trees put together, to classify or predict a particular target with the given set of features/parameters [29]. Each tree in the RF classifier is constructed using ‘randomly’ chosen set of features and ‘randomly’ chosen samples/eyes. This ensures that the classification is repeatable, even when random subsets of eyes/samples were chosen. The classification in each tree is based on the amount of information (entropy) present in the features and interaction of these features with respect to the target variable [29]. The RF classifier then decides the final classification based on the most frequently appeared features (best feature) from multiple trees [29]. The RF classifier was a highly efficient AI classifier for analyses involving images and multiple parameters [29, 30]. Therefore, we used the same to train and cross-validate our AI model using all of the above parameters. The RF classifier consisted of 10 trees and the average of the outcome from the 10 trees was considered (Orange Data Mining software, University of Ljubljana, Slovenia). Leave one out method was used for cross-validation (Orange Data Mining software).Overall the prediction of the RF classifier was defined as the eyes that were accurately classified into one of the 4 groups from the entire dataset of eyes.

### Statistical analyses

The performance of the AI model was evaluated using area under the curve (AUC), classification accuracy (CA), precision (Pr), recall (Rec) and F1-score. The Orange version 3.25.0 data mining package was used for analyses and MedCalc v19 (MedCalc Inc., Belgium) was used for further statistical analyses.

## Results

A total of 300 eyes of 151 patients were analysed, which included 79 males and 72 females with an average age of 29 ± 4.6 years. Thirteen parameters (Table 2) were analysed using the RF classifier. Table 3 shows the parameters in decreasing order of importance as assigned by the RF classifier. Microneuroma was the highest ranked parameter in the RF classifier followed by DC density including (both mature and immature), orthoptic issues (presence or absence) and corneal nerve objective parameters. The CnFrD was the highest ranked nerve parameter followed by CNFL and CNBD. Systemic issues and features like beading of nerves were not selected by the RF classifier among the top six parameters (Table 3).

**Table 3:** Top six corneal nerve and systemic parameters ranked in decreasing order of importance by the artificial intelligence model.

	Parameter	Type
1	Microneuromas	Binary
2	Mature Dendritic Cells	Continuous
3	Immature Dendritic Cells	Continuous
4	Total Dendritic Cells	Continuous
5	Orthoptic related issues	Binary
6	Corneal nerve fibre fractal dimension (CNFrD)	Continuous

Table 4 shows significant associations identified by the RF classifier. The column in the table labelled as ‘number of eyes’ denoted the total number of eyes in the particular group that satisfy the group’s associations compared to the total number of eyes from all groups that satisfy that association. In classifying patients with eye pain, the association of presence or absence of microneuromas with DC and nerve parameters such as CNFrD, CNFL and nerve beading were seen in different combinations across the four groups. Microneuromas were clearly present in majority of eyes (76%) in Group 1 where the discomfort was much more than the signs and was present in a smaller proportion of patients in the remaining three groups.

**Table 4:** Associations identified by a decision tree classifier in the RF AI model.

	Associations	Number of eyes
S^+/-^ D^++^ ^a^	Total dendritic cells>7.3 with immature dendritic cells<83.6, CNFrD ^b^>1.43 and presence of microneuromas	46/72
S^+^ D^+^ ^c^	Total Dendritic cells >7.6 with immature dendritic cells>7 and mature dendritic cell>30.6 with no microneuromas	24/28
S^+^D^-^^d^	Total dendritic cells< 7.3, with CNFrD ^b^ >1.53, no microneuromas and absence of beading of nerves	7/7
S^-^D^-^ ^e^	Total dendritic cells<7.3 and CNFrD ^b^<1.53 with no microneuroma	17/26

^a^ discomfort / symptom grade is higher than the grade/severity of signs

^b^ Corneal nerve fibre fractal dimension

^c^ similar grades of discomfort / symptoms and signs

^d^ without discomfort / symptoms but present with signs

^e^ without discomfort / symptoms and no clinical signs

Hence, microneuromas were an important feature to classify subjects with discomfort greater than signs. The RF model had high accuracy while classifying patients with ocular surface pain. Table 5 shows the accuracy of the RF model for each of the predicted groups. Overall, S^+^D^+^ achieved the highest accuracy while S^+^D^-^ achieved the lowest accuracy. The performance was also evaluated. Over all, the AI achieved an area under the curve of 0.736, accuracy of 86%, F1-score of 85.9%, precision of 85.6% and recall of 86.3%.

**Table 5:** Accuracy of the random forest classifier.

	Predicted	
	Accurately	Not Accurately
S^+/-^ D^++^ ^a^	81.1%	18.9%
S^+^ D^+^ ^b^	86.3%	14.7%
S^+^D^-^ ^c^	69.1%	31.9%
S^-^D^-^ ^d^	79.7%	20.3%

^a^ discomfort / symptom grade is higher than the grade/severity of signs

^b^ similar grades of discomfort / symptoms and signs

^c^ without discomfort / symptoms but present with signs

^d^ without discomfort / symptoms and no clinical signs

## Discussion

Our understanding of ocular surface pain is constantly evolving and possible pathophysiological mechanisms related to ocular surface inflammation, altered nociception or neuropathy were explored [1, 31]. To our knowledge, AI has not been used to understand and quantify the importance of various corneal sub-basal nerve plexus features using confocal microscopy and systemic factors that could be associated with ocular surface pain. Among the various corneal nerve features, microneuromas were detected as the parameter with highest importance by the RF model. Microneuromas are defined as nerve abnormalities present as irregularly shaped, terminal enlargements of sub-basal nerve ending(s) with variable hyperreflectivity. They may occur in the sub-basal nerve plexus or stroma of the cornea [32, 33]. These can be a possible source of discomfort and abnormal sensation in patients especially those with neuropathic corneal pain. This has been attributed to the fact that morphological nerve changes which are seen on confocal analysis led to molecular changes, such as modified protein expressions, which changed the overall excitability of the corneal nerves [32]. This ultimately caused hyperalgesia, allodynia and other symptoms of ocular discomfort [32]. Therefore, these changes were present more in those eyes where there was a disparity between the severity of ocular surface pain or discomfort symptoms (D) and signs (S), especially when symptoms were much greater than the signs noted. In our study microneuromas were present in fifty eight out of seventy six eyes (76%) belonging to group 1 (S^+/-^D^++^) in whom the discomfort/symptoms grade were greater than the grade/severity of signs.

The parameter with next highest importance in the RF model (Table 3) was DC density including both mature and immature. Previous studies have reported an association of DC density with an increased discomfort and ocular inflammation [34, 35]. These DC’s proliferated within a tissue and secreted various inflammatory factors which eventually can lead to raised ocular inflammation and discomfort. There are two types of DC’s: mature and immature. In the presence of even a low-grade ocular surface inflammation, the immature cells start transforming to mature cells [35]. The mature cells were selected by the RF model earlier than the immature dendritic cells in order of decreasing importance highlighting the importance of their association with patients presenting with pain. Interestingly among the associations (Table 4), the total DC count was <7.3 in a large cohort of patients who did not have discomfort (Group 3) compared to those who had discomfort either greater than signs or similar to signs (Groups 1 and 2). Thus, the higher total DC count >7.3 was associated with those with greater discomfort and may be used as a surrogate marker for patients with increased discomfort.

IVCM provided objective parameters to assess corneal sub basal nerve plexus including the CNFL, CNBD, CNBW and CNFrD. The CNFrD is a measure of tissue structural complexity. It has applications in resonance imaging scans where it has been used to identify distinct tissue geometric patterns in different neurodegenerative diseases [36, 37]. CNFrD increased in healthy control subjects. In contrast, fewer, shorter, or disrupted nerve fibers resulted in a lower CNFrD value and indicated altered nerve morphology [38]. CNFrD was the corneal nerve parameter with the highest importance suggesting its’ significant role in detecting corneal nerve changes in patients with ocular surface pain. Earlier studies found that the CNFrD was highly sensitive compared to CNFD, CNBD and CNFL in diagnosing patients with inflammatory neuropathies [38]. This was followed by CNFD and CNFL in order of decreasing importance as reported by the RF model.

Many patients present with non-specific eye pain symptoms and this does pose a major challenge. Interestingly, orthoptic related problems were picked up among the top five parameters (Table 4). Studies have shown that accommodative problems and non-strabismic binocular visual anomalies (NSBVA) due to ever increasing visual demand and use of computers and other screen-based devices can lead to over-exertion of accommodation and convergence [7]. Since the majority of the young population today have a work profile mandating the use of computer, cell phones, or other visual display devices for long hours, this can lead to prolonged use of near and intermediate visual activity [7]. Computer vision syndrome, also known as digital eye strain, includes symptoms that are a result of continuous work in front of the different types of computer screens or other types of digital screens. It includes a wide range of non-specific (asthenopia) symptoms, which include eye fatigue, eye strain, pain in and around the eye, blurred vision, headaches [39]. Among the ocular causes of discomfort and pain that were seen in these patients, accommodative and vergence abnormalities were significantly important factors in addition to dry eyes [39]. Thus, orthoptic related issues were important to consider as a possible differential diagnosis for patients presenting with ocular surface pain. Though systemic factors were not picked up as the top parameters by the RF model, the importance of systemic evaluation and ruling out connective tissue disorders, such as rheumatoid arthritis, systemic lupus erythematosus and Sjogren’s syndrome in patients presenting with ocular surface pain, could arise as a result of undiagnosed dry eye disease [28]. Although studies have suggested that systemic diseases are risk factors for the development of dry eye, they also suggest that these systemic diseases may not be associated significantly with the severity of dry eye as measured by ocular surface examinations and symptom indices and can often be missed through routine evaluation [28]. Hence evaluating for systemic associations and correlating with possible changes in other parameters such as imaging using IVCM can hold clues to possible causes in patients presenting with ocular surface pain.

Corneal nerve beading was also detected by the RF model though not as one of the top six parameters. Beading is neither a specific nor sensitive marker for corneal pain. Beading reflects increased metabolic activity and was seen in normal subjects as well [40]. Another study showed that about 94% of patients with corneal neuropathic pain had beading in their IVCM scans [34]. The assessment and quantification of beading may have value in monitoring patients over time with ocular surface pain [34] though it was not an important corneal nerve feature used by the RF for classification in our dataset and hence was not a sensitive or specific marker while looking at ocular surface pain

A limitation of the present study was that it was done entirely on Asian-Indian population. Another limitation was that the study did not include any measures of change in parameters but looked at a single time point. The purpose of this study was to provide a simple RF framework to combine various IVCM and demographic parameters, which can help us better manage patients presenting with non-specific complaints of ocular surface pain. This is the first study which demonstrates the importance of use of AI in stratification of various parameters in patients presenting with ocular surface pain. However, orthoptic related issues and systemic associations in patients presenting with ocular surface pain should also be considered. The assessment of ocular pain on the basis of questionnaires and correlating it to clinical metrics and confocal features such as corneal dendritic cell density and microneuroma-like structures can help clinicians better understand the condition and aid in customized treatment planning. Treatment which is targeted at improving the nociceptive balance could help improve patient symptoms and long-term comfort. We therefore have proposed an easy-to-use and understand, classification of patients with ocular surface pain using AI, which can help evaluate and stratify patients for customized management. For instance, patients suffering from ocular surface pain (discordant symptoms and signs) with increased corneal dendritic cell density, with or without presence of microneuroma-like structures can be managed with topical anti-inflammatory medications, and may help break the vicious cycle of chronic inflammation driven pain. However, in patients suffering from ocular surface pain (discordant symptoms and signs) with normal corneal dendritic cell density but with presence of microneuroma-like structures can be referred to neurology consult and possibly systemic pharmacotherapy as first line of management strategy. However, it is important that multicentric studies across different ethnicities and larger cohort should be conducted to validate our findings to provide a clinically actionable algorithm that uses these patient specific corneal features for stratifying patients with ocular surface pain.

## Supporting information

S1 File (XLSX)
